# Relationship between albumin-corrected anion gap and short- and medium-term all-cause mortality in heart failure patients with a single ICU admission

**DOI:** 10.3389/fcvm.2025.1608383

**Published:** 2025-08-29

**Authors:** Shaoyan Huang, Qiuwang Zhang, Lei Liu, Michael J. B. Kutryk, Jianzhong Zhang

**Affiliations:** ^1^ Department of Oncology, Yantaishan Hospital Affiliated to Binzhou Medical University, Yantai, Shandong, China; ^2^ Division of Cardiology, Keenan Research Center for Biomedical Science, St. Michael’s Hospital, Unity Health Toronto, University of Toronto, Toronto, ON, Canada; ^3^ Department of Anesthesiology, Yantaishan Hospital Affiliated to Binzhou Medical University, Yantai, Shandong, China

**Keywords:** heart failure, critical care unit, mortality, albumin-corrected anion gap, prognosis

## Abstract

Studies examining the role of albumin-corrected anion gap (ACAG), an emerging promising prognostic biomarker for critical illnesses, in predicting mortality of ICU patients with heart failure (HF) are limited. We aimed to analyze the relationship between ACAG and short- and medium-term all-cause mortality in HF patients with a single ICU admission. Data on HF patients in the Medical Information Mart for Intensive Care-IV (MIMIC-Ⅳ) database were extracted and analyzed. The restricted cubic spline (RCS) model, Kaplan–Meier curve, univariate and multivariate Cox regression, propensity score matching, and mediation analysis were used to assess the association between ACAG concentrations at admission and 30-day and 365-day mortality. Receiver operating characteristic (ROC) curve analysis was performed to evaluate the predictive ability of ACAG for mortality. A total of 4,821 patients were included in this study. The RCS model showed a linear relationship between ACAG and mortality. Based on this result, patients were divided into two groups: ACAG ≥18 mmol/L and ACAG <18 mmol/L. The Kaplan–Meier curve and multivariate Cox regression analysis demonstrated a positive association between ACAG and mortality at both time points. Propensity score matching showed 30-day and 365-day mortality rates in the high ACAG group remained significantly higher compared to the low ACAG group. SAPS II, lactate, BUN, creatinine, and hematocrit partially mediated the association between ACAG and the risk of all-cause mortality. ACAG had an AUC value of 0.647 in predicting mortality. Lactate, the most common and clinically significant unmeasured anion, contributing to ACAG elevation in critical illnesses, was found negatively associated with SpO_2_ and hemoglobin but positively associated with heart rate, ALT, AST, creatinine, and blood urea nitrogen. In conclusion, there is a significant positive association between ACAG and short- and medium-term all-cause mortality in HF patients with a single ICU admission. The ACAG index should be combined with other clinical markers to ensure accurate risk stratification. Clinicians should be cautious in solely relying on ACAG for decision-making.

## 1 Introduction

Cardiovascular diseases are highly prevalent worldwide, with heart failure (HF) representing one of the most significant public health issues (1). HF is characterized by impaired cardiac function, leading to reduced organ perfusion and potential multi-organ damage. It is the leading cause of death and hospitalization in individuals over 60 years old (1, 2). Due to the severity of HF, a large proportion of hospitalized patients require admission to the intensive care unit (ICU) (3, 4). Identifying risk factors for mortality in ICU patients with HF is crucial for optimizing treatment, resource allocation, and a better understanding of the pathophysiology. To date, methods, such as the Acute Physiology Score III (APS III) and the Sequential Organ Failure Assessment (SOFA) score have been explored for assessing disease severity and mortality risk in critically ill ICU patients (5–7). While the SOFA score has been shown to predict long-term all-cause mortality in acute HF patients (5), specific studies focusing exclusively on the application of APS III in HF patients are limited (7). SOFA's complexity, reliance on numerous indicators, and the variability in assessors’ scores are notable shortcomings (8).

Patients with HF often exhibit acid-base and electrolyte disturbances (9, 10). The anion gap (AG), which reflects the difference between the concentrations of measured cations and anions in serum, is a widely used biomarker for acid-base disorders. Tissue hypoperfusion in HF can lead to anaerobic metabolism, increasing the production of organic acids such as lactate (10), a significant unmeasured anion that contributes to AG elevation. Moreover, renal dysfunction, which frequently coexists with HF, impairs clearance and further promotes the accumulation of unmeasured anions. Although elevated AG has been associated with poor prognosis in HF (11–13), fluctuations in albumin concentration (albumin is a major negatively charged plasma protein) can result in underestimation of AG. For every 10 g/L decrease in serum albumin, the AG decreases by approximately 2.3–2.5 mmol/L (14, 15). Given that hypoalbuminemia is common in HF, the albumin-corrected anion gap (ACAG) is particularly relevant for HF patients, as it adjusts for hypoalbuminemia and more accurately reflects the burden of unmeasured anions and acid-base disturbances. ACAG has been investigated as a potential biomarker for disease severity and mortality in various critical conditions, including HF (16–22).

To date, reports on the ACAG role in predicting mortality of ICU patients with HF are limited (21, 22). A study with a small sample size (205 patients with HF) found that ACAG levels were positively associated with mortality in hospitalized HF patients over an average follow-up period of approximately 6 years (21). Li et al. studied 7,787 ICU patients with HF and reported that patients who died at 365 days had significantly higher ACAG levels than survivors, but they did not evaluate ACAG predictive ability for mortality (22). Although both studies identified ACAG as a prognostic marker, they included patients with multiple ICU admissions, who may experience various metabolic derangements. Long-term metabolic adaptation in these cases could affect the prognostic value of ACAG. To clarify its precise role in mortality prediction, studies on HF patients with a single ICU admission are needed. Additionally, both studies examined the relationship between ACAG levels and medium- or long-term (1 year or 6 years) mortality. Since identifying biomarkers for short-term mortality enables early targeted interventions that may help reduce mortality, we aimed to investigate the role of ACAG in predicting 30-day (short-term) and 365-day (medium-term) mortality in ICU patients with HF. To this end, we extracted and analyzed data from the Medical Information Mart for Intensive Care-IV (MIMIC-IV) database. Distinct from the two published studies (21, 22), our current study only selected HF patients with a single ICU admission, and we employed propensity score matching (PSM) to minimize confounding effects and further evaluate the association between ACAG levels and mortality. Additionally, we assessed ACAG's predictive ability for mortality using receiver operating characteristic (ROC) curve analysis.

## 2 Methods

### 2.1 Database and patients

The MIMIC- IV database at the Beth Israel Deaconess Medical Center was used. Data on HF patients with a single ICU admission from 2008 to 2022 were extracted and analyzed. Since this study involved a secondary analysis of anonymized data, which cannot be used to directly or indirectly identify individuals, the requirement for informed consent and additional ethical review was waived. Jianzhong Zhang, one of the authors, obtained access to the database (certification number 66402178).

Diagnostic codes from the 9th and 10th revisions of the International Classification of Diseases (ICD-9 and ICD-10) were used. Patients were included if they had HF with a single ICU admission. Patients with any one of the following conditions were excluded: (1) multiple ICU admissions; (2) age under 18 years, (3) ICU stay of less than 24 h, and (4) absence of albumin or anion gap (AG) data.

### 2.2 Patient variables

The primary outcome measures were mortality at 30 and 365 days. Patient variables upon ICU admission (baseline), including demographic information, comorbidities, laboratory results, vital signs, and severity scores were extracted and analyzed. The variables are as follows: age, gender, respiratory rate, body temperature, heart rate, mean blood pressure (MBP), peripheral oxygen saturation (SpO_2_), comorbidities (diabetes, myocardial infarct, peripheral vascular disease, cerebrovascular disease, chronic pulmonary disease, liver disease, renal disease, and cancer), laboratory data [white blood cell (WBC) counts, hemoglobin, platelet counts, alanine aminotransferase (ALT), aspartate aminotransferase (AST), total bilirubin, albumin, creatinine, blood urea nitrogen (BUN), calcium, chloride, potassium, sodium, potential of hydrogen, lactate, AG, international normalized ratio (INR), prothrombin time (PT), partial thromboplastin time (PTT), alkaline phosphatase (ALP), creatine kinase-MB (CKMB), and lactate dehydrogenase (LDH)], disease severity scores (APS III, SAPS II, Charlson, and SOFA), and medications in the ICU (epinephrine, dopamine, phenylephrine, vasopressin, and norpinephrine). Variables with more than 20% missing values were excluded. Multiple imputation using a random forest-based approach was applied to resolve missing data.

### 2.3 AG and ACAG calculation

The AG was calculated using the following formula: AG = (serum sodium + serum potassium)−(serum chloride + serum bicarbonate), where all electrolytes were measured in mEq/L. ACAG was calculated using the following formula: ACAG = AG + 2.5 × (4.4—serum albumin), where serum albumin was measured in g/dl (14, 15).

### 2.4 Statistical analysis

Normally distributed data were expressed as Mean ± SD and analyzed by Student's t-test or one-way ANOVA with *post hoc* Tukey's analysis. Non-normally distributed variables were presented as median (first quartile, third quartile) and analyzed with Mann–Whitney *U*-test or Kruskal–Wallis test with *post hoc* Dunn's test. Categorical data were presented as percentages and analyzed using Chi-square test or Fisher's exact test.

Various statistical tools were employed to elucidate the relationship between ACAG levels and all-cause mortality, including hazard ratios (HR) and 95% confidence intervals (CI), restricted cubic spline (RCS) curves, PSM, Kaplan–Meier (KM) analysis, and multivariate Cox models, with adjustment for covariates. Mediation analysis was conducted to explore potential pathways through which ACAG may influence all-cause mortality. Variables (and their groupings) were selected for the Cox regression models and mediation analysis based on clinical relevance and prior studies that had evaluated the prognostic value of ACAG in critical illnesses (16–22). Additionally, multicollinearity among the variables was assessed using the variance inflation factor (VIF) analysis. Variables with a VIF greater than 10, indicating a high degree of multicollinearity (Supplementary Table S1), were excluded from RCS, Cox regression models, and mediation analysis. ROC curve analysis was used to evaluate the ability of ACAG to predict mortality. The area under the curve (AUC) value is interpreted as: 0.5 ≤ AUC < 0.6 (failed); 0.6 ≤ AUC < 0.7 (poor or low); 0.7 ≤ AUC < 0.8 (moderate or acceptable); 0.8 ≤ AUC < 0.9 (good); and AUC ≥ 0.9 (excellent) (23, 24). Furthermore, multivariate regression was used to examine factors associated with elevated lactate levels.

All analyses were performed using R Statistical Software (version 4.2.2, http://www.R-project.org, the R Foundation) and the Free Statistics Analysis Platform (version 2.0, Beijing, China, http://www.clinicalscientists.cn/freestatistics). A *p*-value of less than 0.05 from two-tailed tests was considered statistically significant.

## 3 Results

### 3.1 Patient screening

**Figure 1:**
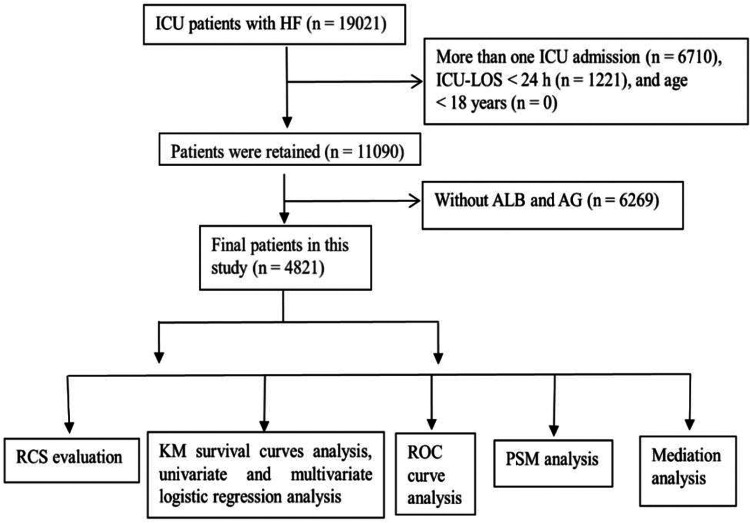
Patient screening flow chart. A total of 19,021 patients underwent screening and 4,821 patients met the criteria and were included in this study.

The patient screening chart is shown in Figure 1. The database contained 19,021 ICU patients with HF. A total of 4,821 patients met the criteria and were included in this study.

### 3.2 Comparisons of baseline variables

The number of patients who died at 30 and 365 days was 1,394 and 2,264, respectively (Table 1). Baseline variables were compared among survivors and patients who died at 30 and 365 days (Table 1). Sex distribution did not differ significantly among different groups of patients. Patients who died were substantially older than survivors and had significantly higher ACAG levels at both time points (Table 1). A higher percentage of patients who died at 30 and 365 days used vasoactive medications, including dopamine, phenylephrine, vasopressin, and norepinephrine, compared to survivors.

**Table 1:** Comparisons of baseline characteristics between survivors and non-survivors.

Variables	30 day mortality			*p*	365 day mortality		*p*
	Total (*n* = 4,821)	Survival (3,427)	Death (1,394)		Survival (2,557)	Death (2,264)	
Gender (Male %)	2,715 (56.3)	1,926 (56.2)	789 (56.6)	0.8	1,456 (56.9)	1,259 (55.6)	0.352
Age (year)	72.6 ± 14.1	71.1 ± 14.4	76.4 ± 12.4	<0.001	69.7 ± 14.7	75.9 ± 12.6	<0.001
Weight (kg)	83.6 ± 26.3	84.8 ± 26.9	80.9 ± 24.6	<0.001	86.6 ± 27.2	80.3 ± 24.8	<0.001
Height (CM)	168.2 ± 10.6	168.7 ± 10.6	167.1 ± 10.4	<0.001	169.4 ± 10.6	166.9 ± 10.4	<0.001
Heart Rate (beats/m)	86.3 ± 17.8	85.4 ± 17.5	88.4 ± 18.3	<0.001	86.0 ± 18.0	86.5 ± 17.5	0.356
MBP (mmHg)	77.5 ± 11.1	78.5 ± 11.3	74.8 ± 10.1	<0.001	79.3 ± 11.4	75.4 ± 10.4	<0.001
RR (breaths/m)	20.5 ± 4.0	20.2 ± 3.8	21.1 ± 4.2	<0.001	20.3 ± 3.9	20.7 ± 4.1	0.006
Temperature (℃)	36.8 ± 0.5	36.8 ± 0.5	36.7 ± 0.7	<0.001	36.9 ± 0.5	36.7 ± 0.6	<0.001
SpO2	96.5 ± 2.3	96.5 ± 2.0	96.3 ± 3.0	0.006	96.5 ± 2.0	96.4 ± 2.6	0.628
Glucose (mg/dl)	156.6 ± 70.1	153.3 ± 68.4	164.6 ± 73.6	<0.001	154.4 ± 69.0	159.0 ± 71.3	0.021
Myocardial infarct (%)	1,699 (35.2)	1,191 (34.8)	508 (36.4)	0.266	877 (34.3)	822 (36.3)	0.145
PVD (%)	727 (15.1)	481 (14)	246 (17.6)	0.001	339 (13.3)	388 (17.1)	<0.001
CVD (%)	788 (16.3)	511 (14.9)	277 (19.9)	<0.001	362 (14.2)	426 (18.8)	<0.001
CPD (%)	1,612 (33.4)	1,120 (32.7)	492 (35.3)	0.081	809 (31.6)	803 (35.5)	0.005
Liver disease (%)	636 (13.2)	409 (11.9)	227 (16.3)	<0.001	292 (11.4)	344 (15.2)	<0.001
Diabetes (%)	1,941 (40.3)	1,368 (39.9)	573 (41.1)	0.446	977 (38.2)	964 (42.6)	0.002
Renal disease (%)	1,835 (38.1)	1,220 (35.6)	615 (44.1)	<0.001	824 (32.2)	1,011 (44.7)	<0.001
Cancer (%)	591 (12.3)	337 (9.8)	254 (18.2)	<0.001	187 (7.3)	404 (17.8)	<0.001
Hematocrit (%)	32.9 ± 6.6	33.3 ± 6.6	32.0 ± 6.5	<0.001	33.8 ± 6.7	31.9 ± 6.5	<0.001
Hemoglobin (g/dl)	10.6 ± 2.2	10.8 ± 2.2	10.2 ± 2.2	<0.001	11.0 ± 2.2	10.2 ± 2.1	<0.001
Platelets (10^9 ^/L)	210.4 ± 107.1	213.1 ± 101.7	203.6 ± 119.2	0.005	215.5 ± 101.0	204.6 ± 113.5	<0.001
WBC (10^9 ^/L)	11.5 (8.4, 15.8)	11.1 (8.2, 15.2)	12.6 (8.9, 17.6)	<0.001	11.2 (8.3, 15.1)	11.9 (8.4, 17.0)	< 0.001
Albumin (g/dl)	3.2 ± 0.6	3.3 ± 0.6	3.0 ± 0.7	<0.001	3.3 ± 0.6	3.1 ± 0.6	<0.001
Anion Gap (mmol/L)	15.6 ± 4.2	15.1 ± 3.8	16.8 ± 4.9	<0.001	15.0 ± 3.8	16.3 ± 4.6	<0.001
Bicarbonate (mmol/L)	22.6 ± 5.1	23.0 ± 4.9	21.8 ± 5.6	< 0.001	23.0 ± 4.7	22.3 ± 5.5	<0.001
BUN (pg/dl)	29.5 (19.0, 47.0)	26.5 (17.5, 42.5)	38.5 (25.0, 57.9)	<0.001	25.0 (17.0, 39.5)	35.5 (23.0, 55.1)	<0.001
Calcium (mg/dl)	8.4 ± 0.8	8.4 ± 0.8	8.4 ± 0.9	0.007	8.4 ± 0.8	8.4 ± 0.8	0.34
Chloride (mmol/L)	101.9 ± 6.7	102.0 ± 6.5	101.7 ± 7.2	0.164	102.2 ± 6.3	101.7 ± 7.1	0.01
Creatinine (mEq/L)	1.4 (0.9, 2.1)	1.2 (0.9, 2.0)	1.6 (1.1, 2.7)	<0.001	1.2 (0.9, 1.9)	1.6 (1.0, 2.6)	<0.001
Sodium (mmol/L)	137.8 ± 5.5	137.8 ± 5.3	138.0 ± 6.1	0.358	137.8 ± 5.2	137.9 ± 5.9	0.778
Potassium (mmol/L)	4.4 ± 0.7	4.3 ± 0.7	4.5 ± 0.7	<0.001	4.3 ± 0.7	4.5 ± 0.7	<0.001
INR (sec)	1.7 ± 1.0	1.6 ± 1.0	1.9 ± 1.1	<0.001	1.6 ± 0.9	1.8 ± 1.1	<0.001
PT (sec)	18.3 ± 10.7	17.4 ± 9.8	20.4 ± 12.2	<0.001	17.0 ± 9.2	19.8 ± 11.9	<0.001
PTT (sec)	44.3 ± 23.8	42.9 ± 22.7	47.7 ± 25.9	<0.001	43.1 ± 23.0	45.6 ± 24.5	<0.001
ALT (u/L)	26.5 (16.0, 59.0)	26.0 (15.5, 52.8)	29.8 (16.0, 75.9)	<0.001	26.5 (16.0, 55.0)	27.0 (15.0, 63.6)	0.455
ALP (u/L)	88.5 (66.0, 125.0)	85.0 (63.5, 119.5)	97.5 (71.0, 141.0)	<0.001	82.0 (62.0, 114.0)	96.0 (70.4, 140.0)	<0.001
AST (u/L)	40.0 (24.0, 94.0)	38.0 (23.5, 81.5)	47.2 (27.0, 132.4)	<0.001	38.0 (24.0, 84.0)	43.5 (25.0, 107.1)	<0.001
Total Bilirubin (mg/dl)	0.7 (0.4, 1.1)	0.7 (0.4, 1.1)	0.8 (0.4, 1.4)	< 0.001	0.7 (0.4, 1.1)	0.7 (0.4, 1.3)	< 0.001
CKMB (u/L)	5.5 (3.0, 14.0)	5.5 (3.0, 13.5)	6.0 (3.0, 15.5)	0.015	5.5 (3.0, 13.5)	6.0 (3.0, 14.0)	0.268
LDH (u/L)	299.0 (223.0, 460.0)	286.0 (215.0, 411.0)	347.0 (251.1, 591.0)	<0.001	283.0 (213.5, 404.0)	323.0 (235.0, 533.1)	<0.001
SOFA	6.0 ± 3.7	5.3 ± 3.4	7.8 ± 3.9	<0.001	5.1 ± 3.3	7.1 ± 3.9	<0.001
APSIII	53.4 ± 21.5	48.3 ± 18.4	65.8 ± 23.2	<0.001	46.7 ± 17.7	61.0 ± 22.7	<0.001
Charlson Index	7.1 ± 2.7	6.7 ± 2.7	8.0 ± 2.6	<0.001	6.3 ± 2.6	8.0 ± 2.6	<0.001
SAPSII	42.8 ± 14.4	39.3 ± 12.7	51.2 ± 14.8	<0.001	38.0 ± 12.3	48.2 ± 14.6	<0.001
Lactate (mmol/L)	1.8 (1.2, 2.7)	1.7 (1.1, 2.5)	2.0 (1.3, 3.5)	<0.001	1.7 (1.1, 2.5)	1.9 (1.2, 3.0)	<0.001
PH	7.4 ± 0.1	7.4 ± 0.1	7.3 ± 0.1	<0.001	7.4 ± 0.1	7.4 ± 0.1	<0.001
Epinephrine, *n* (%)	359 (7.4)	214 (6.2)	145 (10.4)	<0.001	182 (7.1)	177 (7.8)	0.355
Dopamine, *n* (%)	283 (5.9)	145 (4.2)	138 (9.9)	<0.001	102 (4)	181 (8)	<0.001
Phenylephrine, *n* (%)	745 (15.5)	439 (12.8)	306 (22)	<0.001	331 (12.9)	414 (18.3)	<0.001
Vasopressin, *n* (%)	617 (12.8)	247 (7.2)	370 (26.5)	<0.001	175 (6.8)	442 (19.5)	<0.001
Norpinephrine, *n* (%)	1,588 (32.9)	890 (26)	698 (50.1)	<0.001	647 (25.3)	941 (41.6)	<0.001
Hospital-Los (days)	9.7 (5.8, 16.2)	10.2 (6.3, 17.6)	7.8 (4.2, 13.7)	<0.001	9.8 (6.2, 15.9)	9.5 (5.2, 16.5)	<0.001
ICU-Los (days)	3.3 (2.0, 6.3)	3.1 (1.9, 5.7)	4.1 (2.2, 7.8)	<0.001	3.1 (1.9, 5.6)	3.8 (2.0, 7.4)	<0.001
ACAG（mmol/L）	18.5 ± 4.4	17.8 ± 3.9	20.2 ± 5.1	<0.001	17.6 ± 3.9	19.5 ± 4.8	<0.001

Continuous variables are expressed as mean ± standard deviation if normally distributed, or as median (first quartile, third quartile) if not. Categorical variables were presented as *n* (%). RR, respiratory rate; PVD, peripheral vascular disease; CVD, cerebrovascular disease; CPD, chronic pulmonary disease; Los, length of stay.

### 3.3 RCS analysis

**Figure 2:**
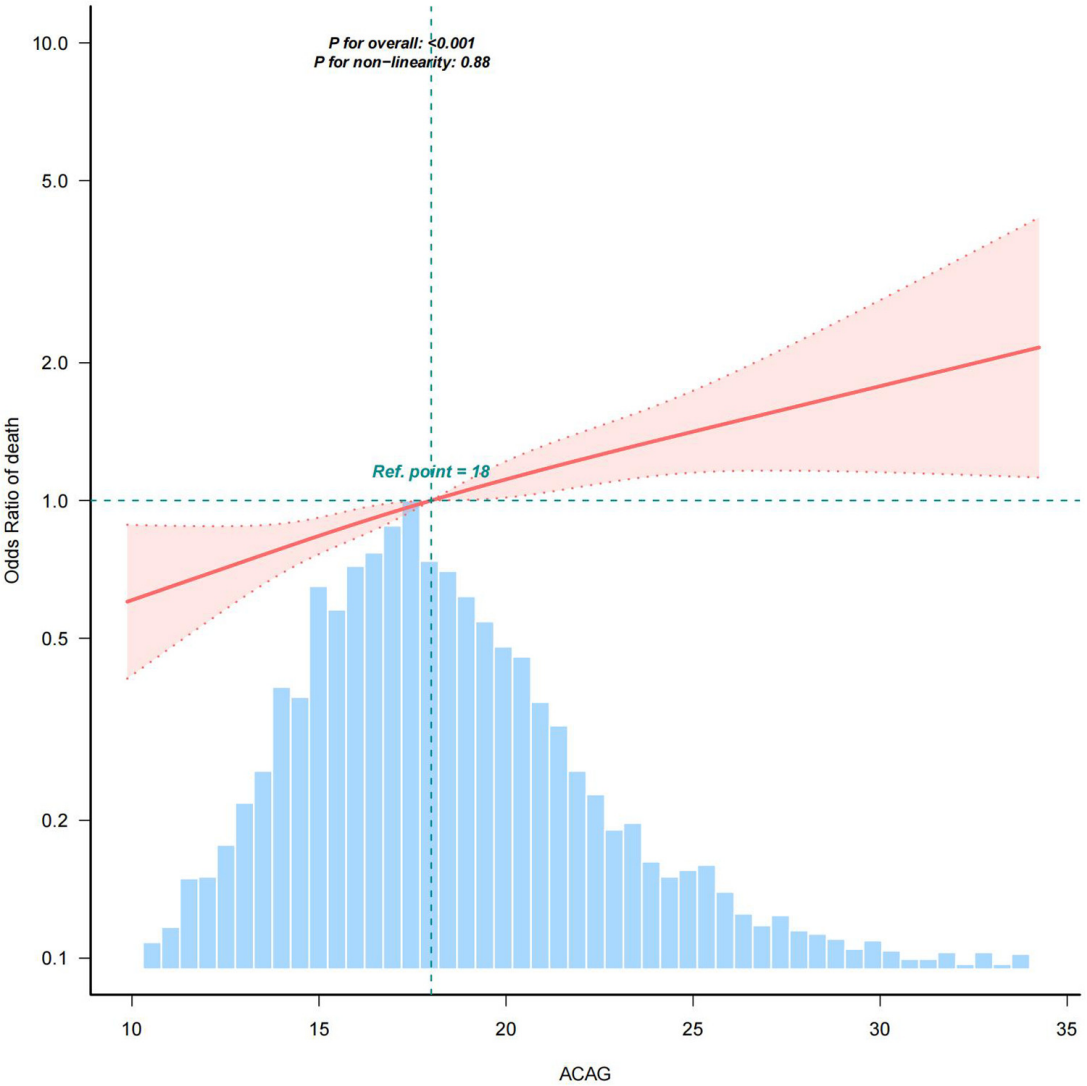
RCS analysis. RCS analysis was performed to assess the association between ACAG and the risk of all-cause mortality in all patients, adjusted for age, gender, heart rate, MBP, respiratory rate, body temperature, SpO2, glucose, diabetes, chronic pulmonary disease, liver disease, renal disease, platelets, hematocrit, BUN, calcium, creatinine, potassium, INR, PT, ALT, AST, CKMB, SOFA, lactate, epinephrine, dopamine, and vasopressin. As shown in this figure, there was a linear association between ACAG and the risk of all-cause mortality. Shaded areas around the red curve depict 99% confidence intervals. The blue histogram represents the distribution of all ACAG values across the study population for which the spline relationship with mortality was modeled.

The RCS curve revealed a linear association between ACAG and the risk of all-cause mortality in all patients (Figure 2). The model using RCS with four knots was fitted within a generalized additive model.

### 3.4 Kaplan–Meier analysis and multivariate Cox regression models

Based on RCS results, ACAG levels were classified into ≥18 mmol/L and <18 mmol/L, and patients were divided into two groups accordingly: ACAGQ1 < 18 mmol/L (*n* = 2,402) and ACAGQ2 ≥ 18 mmol/L, *n* = 2,419). Kaplan–Meier and multivariate Cox regression analyses were performed to evaluate the relationship between ACAG and mortality.

**Figure 3:**
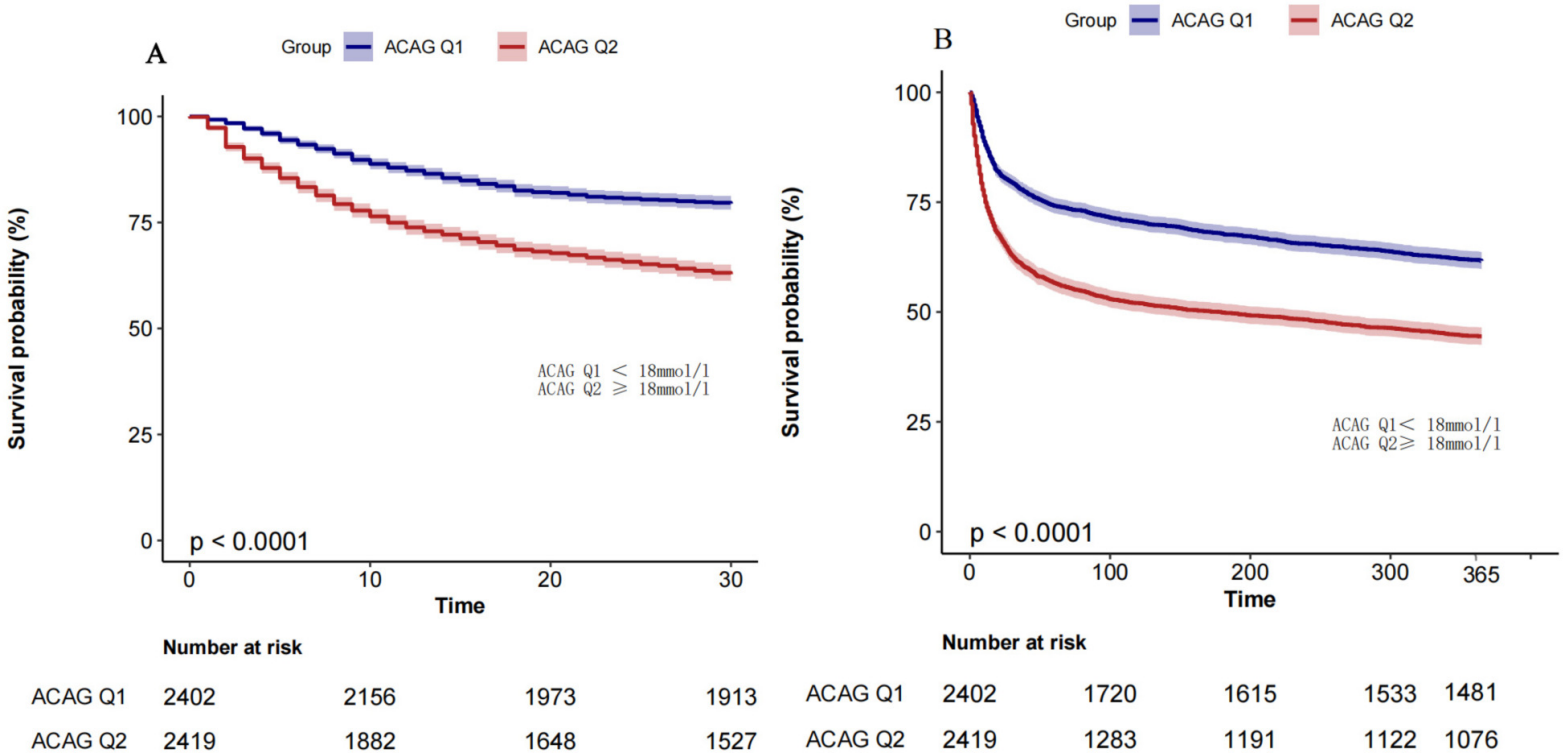
Km analysis. The KM survival curve showed that the 30-day (panel **A**) and 365-day (panel **B**) all-cause mortality rates were significantly higher in ACAGQ2 patients than in ACAGQ1 patients. The *p*-value indicated in this figure is derived from the log-rank test.

As shown by the Kaplan–Meier curve in Figure 3, the survival probability is significantly lower in the ACAGQ2 group than in the ACAGQ1 group at both 30 and 365 days.

Four models were used in multivariable cox proportional hazard analysis. They are (1) the crude model (unadjusted), model I (adjusted for age, heart rate, MBP, respiratory rate, temperature, SpO_2_, and glucose), model II (adjusted for model I + diabetes, chronic pulmonary disease, liver disease, renal disease, platelets, hematocrit, BUN, calcium, creatinine, and potassium), and model III (adjusted for model II + INR, PT, ALT, AST, CKMB, SOFA, lactate, epinephrine, dopamine, and vasopressin). As shown in Table 2, with ACAGQ1 as the reference group, the Cox proportional hazard models showed that the HRs were increased at both 30 and 365 days in the ACAGQ2 group in each model.

**Table 2:** Cox proportional hazard analysis.

Variables	Crude		Model I		Model II		Model III	
	HR (95% CI)	*P* value	HR (95% CI)	*P* value	HR (95% CI)	*P* value	HR (95% CI)	*P* value
30-day								
ACAG mmol/L	1.11 (1.1–1.12)	<0.001	1.09 (1.08–1.1)	<0.001	1.08 (1.07–1.1)	<0.001	1.04 (1.02–1.06)	<0.001
ACAGQ1 < 18 mmol/L	Ref		Ref		Ref		Ref	
ACAGQ2 ≥ 18 mmol/L	2.05 (1.84–2.29)	<0.001	1.65 (1.47–1.85)	<0.001	1.45 (1.29–1.64)	<0.001	1.19 (1.05–1.35)	0.007
365-day								
ACAGQ1 < 18 mmol/L	Ref		Ref		Ref		Ref	
ACAGQ2 ≥ 18 mmol/L	1.75 (1.61–1.9)	<0.001	1.53 (1.4–1.67)	<0.001	1.34 (1.22–1.47)	<0.001	1.14 (1.04–1.26)	0.007

### 3.5 Matched cohort characteristics

PSM was conducted to mitigate selection bias by balancing observed covariates. All relevant variables were included in the propensity score model, except AG, albumin, APSIII, and SAPSII. Matched cohort characteristics are shown in Table 3. After propensity score matching, 30-day and 365-day mortality rates in ACAGQ2 group remain significantly higher than those in the ACAGQ1 group (*p* = 0.043 for 30-day mortality and *p* = 0.029 for 365-day mortality).

**Table 3:** Cohort characteristics before and after propensity score matching.

Variables	Unmatched patients				Propensity Score Matched Patients			
	ACAG (mmol/L)				ACAG (mmol/L)			
N	Total (4,821)	<18 (2,402)	≥18 (2,419)	*p*	Total (2,274)	<18 (1,137)	≥18 (1,137)	*p*
Gender (Male)	2,715 (56.3)	1,351 (56.2)	1,364 (56.4)	0.921	1,247 (54.8)	619 (54.4)	628 (55.2)	0.705
Age (year)	72.6 ± 14.1	72.4 ± 14.1	72.9 ± 14.0	0.148	73.5 ± 13.6	73.4 ± 13.4	73.7 ± 13.8	0.585
Weight (kg)	83.6 ± 26.3	84.4 ± 27.1	82.9 ± 25.5	0.045	83.1 ± 26.4	83.2 ± 25.9	83.0 ± 26.8	0.858
Height (CM)	168.2 ± 10.6	168.5 ± 10.7	168.0 ± 10.5	0.159	167.9 ± 10.4	168.0 ± 10.4	167.8 ± 10.4	0.728
Hear rate	86.3 ± 17.8	83.7 ± 17.1	88.8 ± 18.1	<0.001	86.8 ± 17.6	86.6 ± 17.6	87.0 ± 17.6	0.643
MBP (mmHg)	77.5 ± 11.1	79.0 ± 11.1	76.0 ± 11.0	<0.001	77.0 ± 10.5	77.1 ± 10.1	77.0 ± 10.9	0.85
Respiratory rate	20.5 ± 4.0	20.0 ± 3.8	21.0 ± 4.1	<0.001	20.6 ± 3.9	20.6 ± 3.9	20.7 ± 3.9	0.532
Temperature (℃)	36.8 ± 0.5	36.8 ± 0.5	36.8 ± 0.6	<0.001	36.8 ± 0.5	36.8 ± 0.5	36.8 ± 0.5	0.838
SpO2	96.5 ± 2.3	96.4 ± 2.1	96.5 ± 2.5	0.500	96.5 ± 2.1	96.5 ± 2.1	96.5 ± 2.1	0.981
Glucose (mg/dl)	156.6 ± 70.1	146.3 ± 57.1	166.7 ± 79.7	<0.001	155.3 ± 63.1	154.7 ± 64.8	155.9 ± 61.3	0.634
MI	1,699 (35.2)	795 (33.1)	904 (37.4)	0.002	795 (35.0)	394 (34.7)	401 (35.3)	0.758
PVD	727 (15.1)	346 (14.4)	381 (15.8)	0.192	312 (13.7)	157 (13.8)	155 (13.6)	0.903
CD	788 (16.3)	414 (17.2)	374 (15.5)	0.096	335 (14.7)	149 (13.1)	186 (16.4)	0.029
CPD	1,612 (33.4)	856 (35.6)	756 (31.3)	0.001	778 (34.2)	392 (34.5)	386 (33.9)	0.791
Liver disease	636 (13.2)	259 (10.8)	377 (15.6)	<0.001	284 (12.5)	140 (12.3)	144 (12.7)	0.800
Diabetes	1,941 (40.3)	867 (36.1)	1,074 (44.4)	<0.001	909 (40.0)	451 (39.7)	458 (40.3)	0.764
Renal disease	1,835 (38.1)	691 (28.8)	1,144 (47.3)	<0.001	831 (36.5)	411 (36.1)	420 (36.9)	0.695
Cancer	591 (12.3)	269 (11.2)	322 (13.3)	0.025	294 (12.9)	144 (12.7)	150 (13.2)	0.708
Hematocrit (%)	32.9 ± 6.6	33.8 ± 6.6	32.1 ± 6.5	<0.001	32.9 ± 6.5	32.8 ± 6.4	33.0 ± 6.7	0.567
Hemoglobin (g/dl)	10.6 ± 2.2	10.9 ± 2.2	10.4 ± 2.2	<0.001	10.7 ± 2.2	10.6 ± 2.1	10.7 ± 2.3	0.288
Platelets (10^9 ^/L)	210.4 ± 107.1	205.9 ± 97.9	214.8 ± 115.5	0.004	215.2 ± 105.6	214.6 ± 107.7	215.8 ± 103.5	0.797
WBC (10^9 ^/L)	11.5 (8.4, 15.8)	10.6 (7.9, 14.1)	12.7 (9.0, 17.6)	<0.001	13.2 ± 8.7	13.1 ± 9.9	13.2 ± 7.3	0.788
BUN (pg/dl)	29.5 (19.0, 47.0)	23.5 (16.5, 35.0)	39.0 (24.0, 60.5)	<0.001	33.9 ± 19.7	33.6 ± 19.2	34.3 ± 20.2	0.371
Calcium (mg/dl)	8.4 ± 0.8	8.5 ± 0.7	8.3 ± 0.8	<0.001	8.4 ± 0.7	8.4 ± 0.7	8.4 ± 0.7	0.946
Chloride (mmol/L)	101.9 ± 6.7	102.6 ± 6.5	101.3 ± 6.9	<0.001	102.2 ± 6.5	102.3 ± 6.7	102.2 ± 6.3	0.712
Creatinine (mEq/L)	1.4 (0.9, 2.1)	1.1 (0.8, 1.5)	1.8 (1.1, 3.1)	<0.001	1.6 ± 1.0	1.6 ± 1.0	1.6 ± 0.9	0.307
Sodium (mmol/L)	137.8 ± 5.5	138.2 ± 5.4	137.5 ± 5.6	<0.001	137.9 ± 5.4	137.9 ± 5.6	137.8 ± 5.2	0.701
Potassium (mmol/L)	4.4 ± 0.7	4.3 ± 0.6	4.5 ± 0.8	<0.001	4.3 ± 0.7	4.3 ± 0.7	4.3 ± 0.7	0.651
INR (sec)	1.7 ± 1.0	1.5 ± 0.9	1.8 ± 1.2	<0.001	1.6 ± 0.9	1.6 ± 0.9	1.6 ± 1.0	0.724
PT (sec)	18.3 ± 10.7	16.8 ± 8.3	19.8 ± 12.4	<0.001	17.7 ± 9.3	17.7 ± 9.0	17.8 ± 9.7	0.689
PTT (sec)	44.3 ± 23.8	42.4 ± 23.0	46.2 ± 24.4	<0.001	44.7 ± 24.4	44.7 ± 24.9	44.6 ± 23.9	0.914
ALT (U/L)	26.5 (16.0, 59.0)	24.0 (15.0, 43.9)	31.0 (17.0, 87.8)	<0.001	26.0 (15.5, 53.4)	25.0 (15.0, 50.0)	26.5 (15.5, 60.0)	0.106
ALP (U/L)	88.5 (66.0, 125.0)	82.0 (63.0, 113.0)	95.5 (69.0, 140.0)	<0.001	88.0 (66.0, 123.5)	87.0 (65.0, 122.0)	89.0 (66.5, 126.0)	0.198
AST (U/L)	40.0 (24.0, 94.0)	34.0 (22.5, 64.0)	49.5 (27.5, 145.0)	<0.001	39.0 (24.0, 83.0)	37.0 (23.0, 73.5)	42.5 (25.5, 95.5)	0.001
TB (mg/dl)	0.7 (0.4, 1.1)	0.6 (0.4, 1.0)	0.7 (0.4, 1.4)	<0.001	0.7 (0.4, 1.1)	0.7 (0.4, 1.1)	0.7 (0.4, 1.1)	0.346
CKMB (U/L)	5.5 (3.0, 14.0)	5.0 (3.0, 13.0)	6.0 (3.0, 15.0)	0.009	5.5 (3.0, 13.5)	5.5 (3.0, 14.0)	5.0 (3.0, 12.5)	0.129
LDH (U/L)	299.0 (223.0, 460.0)	275.0 (210.0, 376.0)	333.5 (240.5, 578.2)	<0.001	295.5 (221.0, 432.0)	288.0 (215.0, 426.0)	302.5 (226.0, 444.0)	0.037
SOFA (score)	6.0 ± 3.7	4.9 ± 3.2	7.2 ± 3.8	<0.001	5.8 ± 3.4	5.7 ± 3.3	5.9 ± 3.4	0.196
Charlson (score)	7.1 ± 2.7	6.7 ± 2.7	7.4 ± 2.7	<0.001	7.1 ± 2.7	7.0 ± 2.6	7.2 ± 2.7	0.116
Lactate (mmol/L)	1.8 (1.2, 2.7)	1.6 (1.1, 2.3)	2.0 (1.3, 3.4)	<0.001	2.2 ± 1.4	2.2 ± 1.5	2.2 ± 1.4	0.545
PH	7.4 ± 0.1	7.4 ± 0.1	7.4 ± 0.1	0.116	7.4 ± 0.1	7.4 ± 0.1	7.4 ± 0.1	0.552
Epinephrine	359 (7.4)	135 (5.6)	224 (9.3)	<0.001	149 (6.6)	74 (6.5)	75 (6.6)	0.932
Dopamine	283 (5.9)	87 (3.6)	196 (8.1)	<0.001	123 (5.4)	58 (5.1)	65 (5.7)	0.516
Phenylephrine	745 (15.5)	282 (11.7)	463 (19.1)	<0.001	338 (14.9)	170 (15)	168 (14.8)	0.906
Vasopressin	617 (12.8)	172 (7.2)	445 (18.4)	<0.001	259 (11.4)	126 (11.1)	133 (11.7)	0.644
Norpinephrine	1,588 (32.9)	568 (23.6)	1,020 (42.2)	<0.001	760 (33.4)	376 (33.1)	384 (33.8)	0.722
Los-Hospital (days)	9.7 (5.8, 16.2)	9.2 (5.8, 15.6)	9.9 (5.9, 16.9)	0.065	9.8 (6.0, 16.4)	9.9 (6.0, 16.8)	9.6 (6.0, 15.5)	0.149
Los-ICU (days)	3.3 (2.0, 6.3)	3.2 (1.9, 5.8)	3.6 (2.0, 7.0)	<0.001	3.3 (2.0, 6.0)	3.4 (2.0, 6.1)	3.2 (2.0, 5.8)	0.447
30-day mortality(%)	1,394 (28.9)	494 (20.6)	900 (37.2)	<0.001	633 (27.8)	290 (25.5)	343 (30.2)	0.043
365-day mortality (%)	2,264 (47.0)	921 (38.3)	1,343 (55.5)	<0.001	1,047 (46.0)	488 (42.9)	559 (49.1)	0.029

Continuous variables were expressed as mean ± standard deviation or median (first quartile, third quartile). Categorical variables were presented as *n* (%). MI, myocardial infarction; PVD, peripheral vascular disease; CD, cerebrovascular disease; CPD, chronic pulmonary disease; TB, total bilirubin; Los, length of stay.

### 3.6 Mediation effect analysis

A mediation analysis showed that SAPS II, lactate, BUN, creatinine, and hematocrit partially mediated the association between ACAG and all-cause mortality, accounting for 33.76%, 9.65%, 22.68%, 13.39%, and 5.66% of the mediating effect, respectively (Table 4).

**Table 4:** Causal mediation analysis results.

Pathways	Indirect	95% CI	Direct	95% CI	Mediation	95% CI	*P* value
ACAG→SAPSII→Death	0.0043	0.0034, 0.0053	0.0128	0.0063, 0.0101	33.76%	0.2554, 0.4498	<0.001
ACAG→Lactate→Death	0.0013	2 × 10^−4^, 0.0024	0.0115	0.0097, 0.013	9.65%	0.0157, 0.1935	0.012
ACAG→BUN→Death	0.0029	0.0018, 0.0041	0.0098	0.0076, 0.0115	22.68%	0.1400, 0.3298	<0.001
ACAG→Creatinine→Death	0.0017	4 × 10^−4^, 0.0031	0.0109	0.0088, 0.0128	13.39%	0.0312, 0.2566	0.008
ACAG→Hematocrit→Death	7 × 10^−4^	4 × 10^−4^, 0.0011	0.0119	0.0101, 0.0131	5.66%	0.0303, 0.0885	<0.001

Adjusted: age, heart rate, MBP, respiratory rate, body temperature, SpO_2_, glucose, chronic pulmonary disease, liver disease, diabetes, renal disease, WBC, epinephrine, dopamine, and vasopressin.

### 3.7 ROC curve analysis

**Figure 4:**
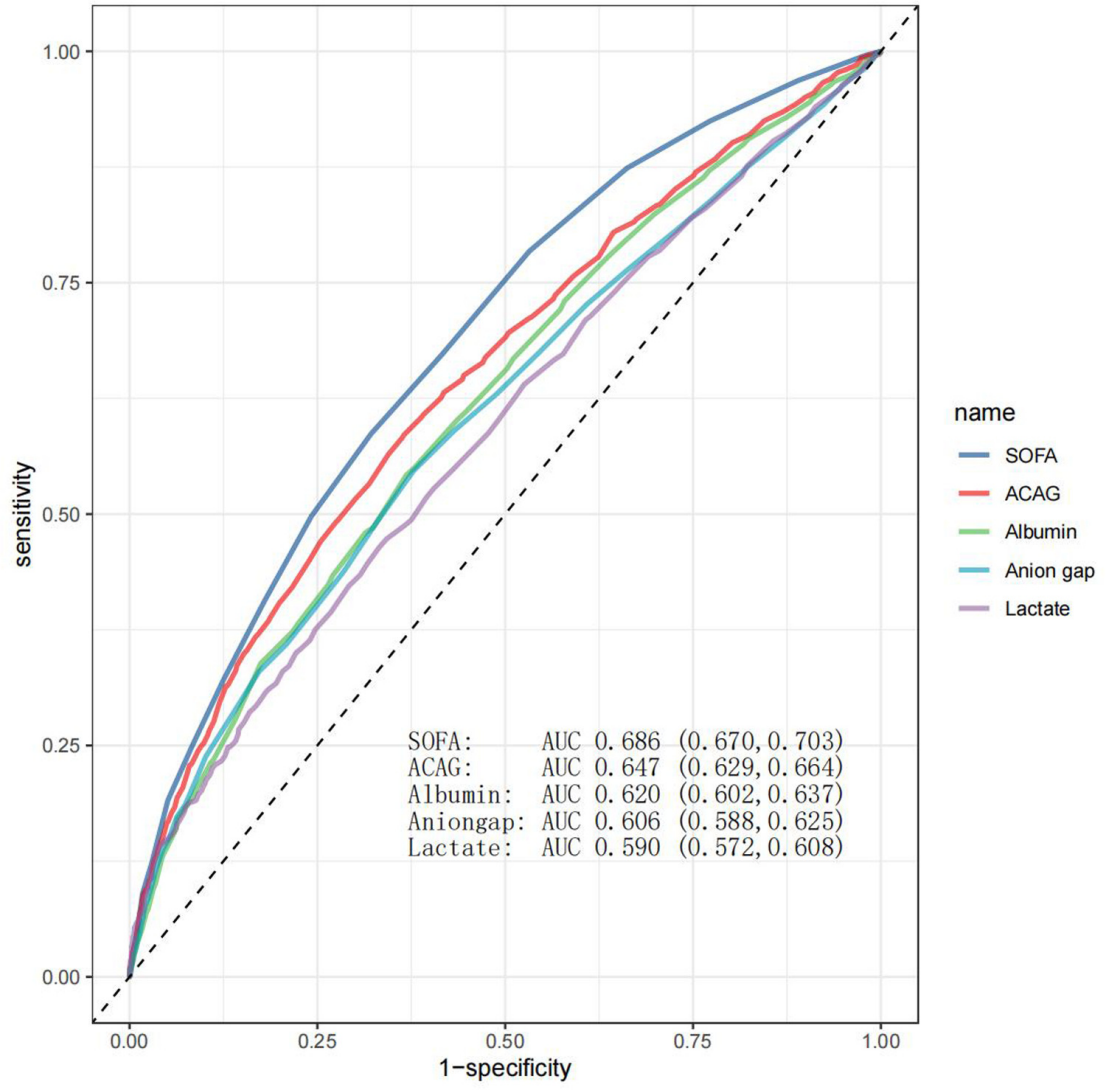
ROC curve analysis. As shown in this figure, SOFA, ACAG, albumin, AG, and lactate had an area under the curve (AUC) value of 0.686, 0.647, 0.620, 0.606, and 0.590, respectively.

The predictive ability of albumin, lactate, AG, ACAG, and SOFA for mortality was assessed by ROC analysis. As shown in Figure 4, ACAG was superior to albumin, lactate, and AG but inferior to SOFA in predicting mortality.

### 3.8 Multivariate regression analysis

Elevated lactate is the most common and clinically significant unmeasured anion, contributing to increased ACAG levels in critical illnesses (25). Multivariate regression analysis revealed that SpO_2_ and hemoglobin were negatively associated with lactate levels, while heart rate, ALT, AST, creatinine, and blood urea nitrogen were positively associated (Table 5).

**Table 5:** Multivariate analysis of factors associated with elevated blood lactate levels.

Variables	B (95% CI)	*P*
Heart rate (beats/m)	0.01 (0.01, 0.02)	<0.001
SpO2 (%)	−0.04 (−0.06, −0.02)	0.001
Hemoglobin (g/dl)	−0.03 (−0.06, −0.01)	0.01
ALT (unit/L)	0.02 (0.01, 0.03)	<0.001
AST (unit/L)	0.01 (0.01, 0.02)	<0.001
LDH (unit/L)	0.01 (0.01, 0.02)	<0.001
Creatinine (pg/dl)	0.02 (0.01–0.03)	0.03
Blood urea nitrogen (mEq/L)	0.03 (0.01–0.07)	0.02

## 4 Discussion

In this study, we focused on patients with a single ICU admission. This selection aimed to recruit a relatively homogeneous cohort to reduce confounding factors related to multiple admissions, such as varying interventions, chronic complications, and changes in clinical status over time, which could obscure the direct relationship between ACAG and outcomes. By analyzing the physiological derangements reflected by ACAG at ICU admission and their impact on prognosis, especially 30-day mortality, while minimizing confounding, we aimed to elucidate the actual role of ACAG as a prognostic biomarker in the group of patients we chose to study. We present the following findings: (1) patients who died at 30 and 365 days had significantly higher ACAG levels compared to survivors; (2) ACAG levels showed a positive association with mortality at both time points; (3) propensity score matching confirmed a positive relationship between ACAG levels and mortality; (4) SAPS II, lactate, bicarbonate, BUN, creatinine, and hematocrit indirectly mediated the association between ACAG and all-cause mortality; (5) ACAG had a higher AUC value than AG in predicting mortality; and (6) SpO₂ and hemoglobin were negatively associated with lactate levels, while heart rate, ALT, AST, creatinine, and blood urea nitrogen were positively associated.

Identifying high-risk ICU patients with HF within 30 days enables urgent interventions, such as intensive monitoring, medication adjustments, and advanced therapies, to reduce mortality. Additionally, many HF patients are readmitted to ICU within 30 days (26), and biomarkers predicting early mortality can guide post-discharge management to reduce the risk of readmission. Therefore, we first compared ACAG levels between patients who died at 30 days and survivors. Our results showed that patients who died at 30 days had significantly higher ACAG levels that were positively associated with mortality. To our knowledge, this is the first report on ACAG predicting short-term mortality in ICU patients with HF.

A few studies have investigated the relationship between ACAG levels and mortality in patients with HF (21, 22). In a retrospective, real-world study of hospitalized HF patients (whether admitted to ICU was not specified), it was found that the death group had significantly higher baseline ACAG levels than the survival group during a mean follow-up period of ∼6 years (21). Another study involving over 7,500 ICU patients with HF reported that ACAG was associated with higher 1-year all-cause mortality (22). This 1-year data, along with ours, suggest a positive association between ACAG levels and short- and medium-term mortality in ICU patients with HF. Compared to the two previous studies (21, 22), which relied solely on multivariate Cox regression, our study employed a more rigorous approach by combining propensity score matching with Cox regression to more effectively minimize confounding effects. Our results confirmed a positive association between ACAG levels and mortality after propensity score matching.

In the present study, SAPS II and BUN were found to be the most significant indirect mediators of the association between ACAG and all-cause mortality, as shown by mediation effect analysis. The SAPS II scoring system incorporates both acute physiological parameters and chronic health conditions, making it a more comprehensive tool for ICU mortality prediction (27). In contrast, the APS III scoring system exclusively evaluates acute physiological parameters to assess the severity of acute illnesses (28). Therefore, SAPS II was selected as a key variable for mediation analysis. It is well documented that comorbidities relate to all-cause mortality in HF patients (29–31). The European Heart Failure Pilot Study showed that diabetes, chronic kidney disease, and anemia were independently associated with increased mortality in HF patients (29). Another study from the UK found that diabetes, chronic kidney disease, and lung disease substantially increased the risk of mortality in HF patients (30). A recent systematic review and meta-analysis study also revealed that diabetes and chronic kidney disease were significant risk factors for mortality in HF patients (31). These previous findings align with the mediating role of SAPS II in the association between ACAG and mortality observed by our study. Therefore, identifying comorbidities in heart failure and initiating prompt, effective treatment is crucial for improving outcomes. We found that BUN, a key marker of kidney function, accounted for 22.68% of the mediated effect. Higher baseline BUN has been reported as a strong predictor of increased post-discharge mortality in patients hospitalized for HF (32), supporting our finding that BUN significantly mediated the association between ACAG and mortality.

Elevated lactate is the most common and clinically significant unmeasured anion, contributing to increased ACAG levels in critical illnesses (25). Lactate accumulation can occur due to tissue hypoperfusion in the context of HF (33). The liver and kidneys are two primary organs responsible for lactate clearance (34). Based on these considerations, heart rate, SpO_2_, hemoglobin, ALT, AST, creatinine, and blood urea nitrogen were included in the multivariate regression analysis to examine their associations with lactate levels. The results showed that SpO_2_ and hemoglobin were negatively associated with lactate levels, while heart rate, ALT, AST, creatinine, and blood urea nitrogen were positively associated. These findings suggest that tissue hypoxia caused by HF promotes anaerobic metabolism and increases lactate production, while liver and kidney dysfunction impairs lactate metabolism and excretion, ultimately leading to lactate elevation and contributing to increased ACAG levels. This is also reflected in our finding that lactate mediated the association between ACAG and the risk of all-cause mortality. Therefore, optimizing oxygen delivery through increasing hemoglobin levels, maintaining sufficient SpO_2_, close monitoring and support of hepatic and renal function are critical to reduce lactate levels and improve outcomes in patients with HF. In the present study, the predictive ability of lactate for mortality was found to be inferior to that of ACAG. While lactate levels were associated with lower SpO_2_ and hemoglobin, and higher ALT, AST, creatinine, and blood urea nitrogen, we did not analyze the extent to which lactate contributes to the observed increase in ACAG, which could be an interesting direction for future research.

A previous study assessed the ability of ACAG to predict long-term mortality (approximately 6 years) in hospitalized HF patients, and reported an AUC value of 0.773, indicating a moderate or acceptable level of performance (21). However, these authors studied a small sample size (205 patients) and did not provide details on hospitalization frequency. We found an AUC value of 0.647 for ACAG, which fell within the category of poor or low performance, although it was higher than that of AG. Our findings highlight the limited standalone predictive value of ACAG, support its use alongside other clinical markers to improve prognostic accuracy, and may alert other clinicians and researchers to avoid redundant studies.

This study has the following limitations: (1) it is observational, limiting the ability to infer causality; (2) ACAG data were missing in approximately 40% of patients, which, although likely random, may still introduce bias and reduce statistical power; (3) the data analyzed in the present study were collected from a single medical center, limiting the generalizability of the findings; and (4) as MIMIC-IV includes data only from the Beth Israel Deaconess Medical Center, prior ICU admissions at other institutions could not be accounted for and may represent a source of unmeasured confounding. Prospective studies are needed to validate our findings.

## 5 Conclusion

There is a significant positive association between ACAG and short- and medium-term all-cause mortality in HF patients with a single ICU admission. Notably, according to ROC analysis, ACAG had a performance that ranges from poor to, at most, moderate, in predicting mortality. The ACAG index should be combined with other clinical markers to ensure accurate risk stratification, and clinicians should be cautious in solely relying on ACAG for decision-making. Taken together, if anything, our results reaffirm that pathophysiologically uninformed composite scores, when used in isolation, are unlikely to yield strong predictive ability, and that it is questionably whether this meaningfully informs therapeutic intervention targets.

## Data Availability

The datasets presented in this study can be found in online repositories. The names of the repository/repositories and accession number(s) can be found below: https://mimic.mit.edu/.
