# Supplementary material for: Relationship between albumin-corrected anion gap and short- and medium-term all-cause mortality in heart failure patients with a single ICU admission
Source: Front Cardiovasc Med. 2025 Aug 29;12:1608383. doi: 10.3389/fcvm.2025.1608383 (PMC12426248; doi:10.3389/fcvm.2025.1608383)
Supplement: Supplementary file 1 [file Table1.docx]

Supplementary Table 1. The variance inflation factor (VIF) of variables

| Variables | VIF |
| --- | --- |
| Age | 1.252 |
| Gender | 1.687 |
| Hear rate | 1.279 |
| MBP | 1.259 |
| Respiratory rate | 1.229 |
| Temperature | 1.145 |
| Sp02 | 1.125 |
| Glucose | 1.284 |
| Diabetes | 1.257 |
| Chronic pulmonary disease | 1.038 |
| Liver disease | 1.126 |
| Renal disease | 1.428 |
| WBC | 2.389 |
| Platelets | 1.154 |
| Hematocrit | 1.238 |
| Hemoglobin | 3.236 |
| BUN | 2.079 |
| Chloride | 1.962 |
| Sodium | 1.762 |
| Calcium | 1.207 |
| Creatinine | 2.316 |
| Potassium | 1.242 |
| INR | 8.151 |
| PT | 8.531 |
| PTT | 6.712 |
| AST | 1.322 |
| ALT | 1.089 |
| CKMB | 1.09 |
| SOFA | 1.833 |
| SAPS III | 2.398 |
| SAPS II | 1.326 |
| Charlson Index | 1.658 |
| Lactate | 1.687 |
| Epinephrine | 1.272 |
| Dopamine | 1.088 |
| Vasopressin | 1.419 |
